# Hypertensive Disorders of Pregnancy Deaths: A Four-Year Review at a Tertiary/Quaternary Academic Hospital

**DOI:** 10.3390/ijerph22070978

**Published:** 2025-06-20

**Authors:** Zeenat L. Khan, Gaynor M. Balie, Lawrence Chauke

**Affiliations:** 1 Department of Obstetrics and Gynaecology, School of Clinical Medicine, Faculty of Health Sciences, University of the Witwatersrand, Johannesburg 2193, South Africa; zeenatlenina@gmail.com (Z.L.K.); gaynor.balie16@gmail.com (G.M.B.); 2 Gauteng Department of Health, Charlotte Maxeke Johannesburg Academic Hospital, 5 Jubilee Road, Parktown, Johannesburg 2193, South Africa

**Keywords:** hypertensive disorders of pregnancy, maternal mortality, South Africa, avoidable factors, eclampsia, quality of care

## Abstract

Background: Hypertensive disorders of pregnancy (HDPs) are a major cause of maternal morbidity and mortality worldwide. Very little progress has been made in reducing HDP-related maternal deaths in low- and middle-income countries (LMICs), including South Africa, over the past decade. Aim: The aim of this study was to describe maternal deaths arising from HDPs at tertiary/quaternary hospital in Johannesburg, South Africa, with specific focus on maternal characteristics, management, timing of death, causes, and avoidable factors and to use the information to inform clinical practice. Methods: We conducted a retrospective review of patient clinical records covering the period 1 January 2015 to 31 December 2018. Data on maternal demographic and pregnancy characteristics, management, causes, and timing of death were extracted from the clinical records and transferred into a Microsoft Excel^®^ Spreadsheet and analysed using descriptive statistics. Results: During the study period, 70 maternal deaths were recorded, of which 23 (32.8%) were due to HDP-related complications. The majority of the maternal deaths, 20 (86.9%), occurred during the postpartum period, predominantly affecting Black African women, 23 (100%), with a median age of 27 years. Notably, 18 (78.2%) of the deceased had booked early and attended antenatal care (ANC). Eclampsia emerged as the most common final cause of death. Key avoidable factors included non-adherence to established protocols, particularly failure to initiate aspirin prophylaxis in at-risk women, as well as incorrect or inadequate administration of antihypertensive therapy and magnesium sulphate (MgSO_4_) prophylaxis. Conclusions: HDP-related maternal deaths are largely preventable. They primarily result from poor quality of care due to a lack of adherence to evidence-based protocol.

## 1. Introduction

While significant progress has been made in reducing maternal mortality over the past decade globally, the number of women who continue to succumb to pregnancy-related complications each year remains unacceptably high [1]. Approximately 99% of these deaths occur in low- and middle-income countries (LMICs), and Sub-Saharan Africa (SSA) accounts for approximately 66% of the global burden of maternal deaths [2,3]. Hypertensive disorders of pregnancy (HDPs) are among the leading direct causes of maternal mortality, accounting for 14% of maternal deaths worldwide [3]. The global estimated incidence of HDPs ranges from 4 to 25%, with higher incidence reported in LMICs [4].

The number of women who lose their lives each year due to complications of HDPs in LMICs, including South Africa, is also unacceptable [1,5]. For many years, non-pregnancy-related infections (NPRIs), hypertensive disorders of pregnancy (HDPs), obstetric haemorrhage (OH), medical and surgical disorders (M&S), and pregnancy-related sepsis (PRS) have ranked among the top five causes of maternal mortality in South Africa (SA) [4,5]. Although maternal deaths due to NPRIs and OH have decreased, the reduction in HDP-associated maternal deaths remains sub-optimal [5,6].

The situation described above is not unique to SA but common to many African countries. Compared to other regions globally, Africa has the highest age-standardised prevalence of hypertension, affecting 46% of its adult population over the age of 25 [7]. Preeclampsia is more commonly observed at the extremes of age, with the highest incidence reported among individuals under 20 and over 35 years of age [8]. Nulliparous, obese, and women carrying multiple gestations are at the greatest risk [9]. Eclampsia and pre-eclampsia are the most common forms of HDPs commonly associated with maternal deaths in LMICs, with pulmonary oedema, renal failure, respiratory failure, and intracranial haemorrhage being the most common final causes of deaths in these cases [6,7,8,9,10,11].

Like other maternal deaths resulting from direct pregnancy complications, the majority of HDP-related maternal deaths can be prevented with existing, cost-effective, and evidence-based interventions that are accessible to low- and middle-income countries (LMICs). Between 2016 and 2019, it is noteworthy that 62.4% of hypertensive disorder of pregnancy (HDP)-related maternal deaths in South Africa were classified as potentially avoidable [6,10,11]. This percentage increased to 70.6% during the 2019–2022 triennial period. Importantly, a considerable number of women who succumbed to complications of HDPs attended antenatal care [5,6,10,12]. This observation highlights underlying concerns regarding the quality of care provided, particularly, inadequate patient assessment and insufficient problem recognition at community healthcare clinics (CHCs) and district hospitals (DHs) [5,6]. The situation is further compounded by non-compliance with established guidelines and protocols at DHs, regional hospitals (RHs), and tertiary hospitals (THs) [5,6,8,11,12].

The most common healthcare system-related factors contributing to avoidable maternal deaths due to complications of HDPs in South Africa include the inappropriate management of high blood pressure (BP), a failure to identify at-risk women, incorrect triage, delays in seeking specialist consultation, and a lack of or delay in emergency transport, which hinder timely referrals to higher levels of care during obstetric emergencies [12]. Additionally, administrative challenges such as overcrowding in healthcare facilities, inadequate resources (both infrastructural and human), medication shortages, and a lack of high care and intensive care unit (ICU) beds further exacerbate these issues [5,8,11,12].

The burden of HDP-related maternal deaths is further exacerbated by socio-economic challenges in SA and other LMICs. Factors such as cross-border migration, limited education, inadequate health literacy, and poverty significantly contribute to maternal vulnerability [6]. The negative experiences of pregnant migrant women accessing maternity services in South Africa have generated significant debate, particularly concerning issues of overcrowding and competition for scarce resources with local populations, juxtaposed with the universal rights of migrants to access healthcare [13].

To address these issues, public health interventions, including public education, community-based screening programmes, and health promotion, are essential [6,7]. Beyond clinical management, preventing HDP-related maternal deaths requires a comprehensive public health response. Community-based screening programmes for hypertension and early pregnancy identification can facilitate timely risk stratification and referral [6,7,8,10,11]. Strengthening primary healthcare systems to ensure adherence to national antenatal guidelines, including the routine provision of low-dose aspirin for high-risk women, is critical [6,8]. Public health education campaigns targeting women of reproductive age could enhance awareness of HDP symptoms and the importance of antenatal care, particularly in underserved communities [5,12]. These population-level strategies, that include the promotion of family planning [14], are integral to achieving sustainable reductions in maternal mortality due to HDPs in South Africa and similar contexts.

There is a paucity of facility-level data from South African tertiary/quaternary institutions that provide detailed analysis of HDP-related maternal deaths. This study addresses this critical gap by providing a review of maternal deaths due to HDP-related complications over a 4-year period. Specifically, the study examined the profile of the women who died, management practices, timing of deaths, causes, and avoidable factors with the aim of using the information to inform practice. Focusing on specific diseases contributing to maternal mortality has the potential to improve our understanding of the underlying factors, including guiding targeted interventions.

## 2. Materials and Methods

### 2.1. Study Design

This is a retrospective cross-sectional study based on the review of the clinical records of women who died of HDP-related complications at a tertiary/quaternary academic hospital in Johannesburg Gauteng Province, South Africa, over a four-year period (from the 1 January 2015 to the 31 December 2018).

### 2.2. Context

This study took place at Charlotte Maxeke Johannesburg Academic Hospital (CMJAH). The site is one of South Africa’s ten central hospitals, specifically a tertiary/quaternary academic hospital located in Johannesburg, Gauteng Province, South Africa. Gauteng Province is South Africa’s economic hub. Although Gauteng is one of the smallest provinces by land area, it has the highest population density, with an estimated population of 16 million [15]. CMJAH has 1088 beds, of which 106 (9.7%) are designated as obstetric beds, comprising emergency obstetric admissions, antenatal care, obstetric high care, labour ward, and postpartum care beds. These figures exclude gynaecology beds. This facility functions as a referral centre for several healthcare institutions that include six regional hospitals (RHs), four district hospitals (DHs), and over ten community healthcare centres (CHCs). Additionally, the hospital accepts tertiary and quaternary referrals from neighbouring provinces such as Limpopo, Mpumalanga, and Northwest. The maternity unit delivers between 9000 and 11,000 women annually, with 33% of the patients coming from neighbouring Southern African Development Community (SADC) countries, the majority of whom are from Zimbabwe.

### 2.3. Study Sample

The study sample comprises all recorded HDP-related maternal deaths that occurred at the study site over a four-year period (from 1 January 2015 to 31 December 2018).

### 2.4. Data Collection and Analysis

All maternal deaths occurring at the institution are reviewed within 24 h of their occurrence. The discussion centres on the causes and avoidable factors, with the information utilised to inform targeted interventions. The Department of Obstetrics and Gynaecology at the study site maintains records and summaries of these discussions, and the deaths are reported to the provincial Maternal, Child, Neonatal, and Women’s Health (MCNWH) Directorate for inclusion in the national maternal death surveillance programme.

The files of the deceased women were obtained from the hospital Patient Records Department. The following data was extracted from the patient clinical files and maternal mortality audit: maternal demographic and pregnancy information, causes of death, and management details. This data was extracted onto a data collection sheet specifically designed for the study and subsequently exported to an Excel Spreadsheet *^®^*2019 for statistical analysis. Descriptive statistics, including frequencies, medians, interquartile ranges (IQRs), and ranges, were calculated to characterise the data.

### 2.5. Ethics

Permission to conduct this study was granted by the Chief Executive Officer (CEO) at the study site, and ethics clearance was obtained from the Human Research Ethics Committee (HREC) of the University of the Witwatersrand (ethics approval number: M200765).

Patient consent was not required because of the retrospective nature of the study.

## 3. Results

### 3.1. Maternal Demographics, Pregnancy Characteristics, and Initial Management

Table 1 is a summary of the profile of the women who died. There were 70 maternal deaths during the four-year study period and 23 (32.8%) were due to HDP-related complications. All the women who died were Black African, 23 (100%). The median age and parity of the study population at the time of death was 27 and one, respectively. Out of the 23 women who died, 18 (78.2%) had booked and attended antenatal care (ANC) clinics. The median numbers of antenatal care clinic visits were three, and 9 out of the 18 (50.0%) attended at least four ANC visits. The median estimated gestational age (EGA) at booking was 15 weeks. The women booked with median systolic blood pressure (SBP) of 135 mmHg and median diastolic (DBP) blood of 79 mmHg.

The majority of the women, 17 (73.9%) were HIV-negative. Of the six women (26.1%) who tested positive for HIV, the median CD4 count was 157. Only two women (33.3%) had an undetectable viral load. There were eight (34.8%) women with at least one risk factor for HDPs. Information to calculate BMI was only available for eight (34.8%) women, and seven (87.5%) out of the eight were classified as overweight to obese. The median BMI was 33.5 kg/m^2^. In total, eight (34.8%) women had co-morbidities (some more than one): three (37.5%) had chronic hypertension (CHT), one (12.5%) Diabetes Mellitus (DM), and four (50.0%) gestational hypertensions (GHT) in the previous pregnancy. Low-dose aspirin was initiated in only one (12.5%) of this group of women with risk factors.

### 3.2. Management at Presentation at Referral Facilities and Study Site

The management of the women at the referring and receiving healthcare facilities (study site) is summarised in Table 2. There was a total of eighteen (78.3%) referrals, nine (50%) from DHs and CHCs, eight (44.41%) from RHs, and one (5.6%) from tertiary-level hospitals. The median SBP and DBP when the women presented at the referring healthcare centres were suggestive of acute severe hypertension with SBB and DBP of 160 and 110 or more, respectively. All had proteinuria and thus were presumed to have pre-eclampsia. Other common symptoms of severity were symptoms of imminent eclampsia recorded in nine (50.0%) and seizures in three (16.7%). Twelve (66.7%) out of the eighteen women required acute management of elevated blood pressure before transfer; however, only five (41.7%) received such treatment. Furthermore, only ten (83.3%) and seven (58.3%) received magnesium loading and maintenance doses, respectively. Long-acting anti-hypertensives were given to nine (50%) of the women only.

The nearest referring healthcare facility is 3 km away and the furthest approximately 48.3 km from CMJAH. The median interval between referral and arrival time from referring healthcare facilities to the study site was 117.5 min, with the longest interval being 672 min. In all cases, the delays were due to either non-availability or delay in emergency transport or ambulances. Only one woman arrived at the study site within 30 min of referral.

The median SBP and DBP during admission to the study site were 149.5 mmHg and 103.5 mmHg, respectively. These values were much lower than those reported at the referring healthcare centres. This might be due to the fact that some of the women received acute antihypertensive therapy with or without magnesium sulphate before transfer. However, a total of 16 (69.6%) women had elevated blood pressure requiring acute management on arrival at CMJAH, but only 12 (75.0%) out of the 16 received treatment, with 7 (43.8%) receiving oral calcium channel blocker Nifedipine^®^ and 5 (31.3%) alpha-beta blocker Labetalol^®^.

The majority of the women, 16 (69.6%), were managed in the obstetric high care unit (OHCU) or ICU and the remainder in normal ward. In total, only 15 (65.2%) of the 23 patients received long-acting first-line antihypertensive treatment, with a further 4 (17.4%) and 2 (8.7%) receiving second- and third-line long-acting agents, respectively. Loading and maintenance doses of magnesium sulphate were given to 13 (81.2%) and 9 (56.3%) patients, respectively.

Pregnancy outcomes and maternal complications are summarised in Table 3. Twenty (87.0%) out of the women who died were delivered before they died, sixteen (80.0%) via emergency caesarean section and four (20.0%) through induction of labour. The indications for emergency caesarean section were HELLP syndrome reported in 15 (93.8%) and suspected foetal distress in the remaining 1 patient. All the caesarean sections for HELLP syndrome were performed under platelet cover, with the lowest and highest platelet counts at 31 and 66. The median gestational age at delivery was 32 weeks.

Regarding maternal complications, most of the women, 17 (73.9%), had renal dysfunction, followed by HELLP syndrome in 15 (65.2%). The remainder of the complications included eclampsia in nine (39.1%), intracranial haemorrhage in four (17.4%), pulmonary oedema in three (13%), and subcapsular haematoma in two (8.7%). Some of the women had more than one complication. Eclampsia and renal failure were the leading causes of death accounting for six (26.1%) of the maternal deaths each, followed by HELLP syndrome with DIC in four (21.7%) and intracranial haemorrhage in four (17.4%). The remaining two (8.7%) women died of pulmonary embolism post-caesarean section. Cardiopulmonary resuscitation (CPR) was performed on 16 (76.2%) women. The prognosis was poor for one woman, while another was determined to be brain dead; hence, neither was resuscitated. The reasons for not performing CPR in the remaining five women were not clear. In terms of timing of deaths, 20 (87.0%) occurred during the postpartum period and the remaining 3 (13.0%) during the antenatal period. Of the maternal deaths, 12 (52.2%) occurred in ICU and 7 (30.4%) in HCU. The median day of death was four days post-delivery. A total of 21 (91.3%) women who died had a postmortem examination which was used to assign the final cause of death.

All maternal deaths were analysed for avoidable factors in three areas: patient-related, administrative, and healthcare-related. The most common avoidable factors included delays or lack of transport in 17 cases (73.9%) and inappropriate management or response to abnormal clinical findings in 12 cases (52.2%). Additionally, patient-related factors, particularly the lack of antenatal care and late or inadequate attendance for antenatal care, were identified in 47.5% of cases (Table 4).

## 4. Discussion

In this study, we described the profile of women who died of HDP-related complications, their clinical management, causes of deaths, and avoidable factors over a four-year period at the study site. The majority of the women were young, highlighting the devastating effect of HDPs in society. This is in contrast with the South African Saving Mothers Reports [5,8,11] and a study by Wang et al. [2], both of which found women at extremes of age (less than 20 and more than 35 years old) to be at the highest risk of HDP-related maternal deaths and HDPs in general. Similarly, a Norwegian study by Nyflot et al. reported that women who had died from HDPs were younger compared to those who had died from other causes [16]. Maternal deaths have substantial economic and social consequences. These consequences include an increased risk of neonatal and infant mortality, behavioural challenges, school dropout rates, and early marriage among orphaned offsprings, as well as social and financial instability for families that have lost a caregiver [17].

The median parity of our study population was one. This is in contrast to a study conducted in Western Saudi Arabia, which reported a higher incidence of HDPs among multigravid women, accounting for 56.7% of their study population [18]. These discrepancies may be attributed to genetic and environmental factors. The majority of the women who died in our study had no identifiable risk factors; however, this finding should be interpreted with caution, as body mass index (BMI) was calculated for only eight patients due to insufficient information. A study analysing changes in maternal mortality rates (MMRs) indicated that rising obesity rate was a major contributing factor to HDP-related maternal mortality [19]. The majority of women tested HIV-negative.

The ideal transfer time during an obstetric emergency is between 30 min and 2 h, depending on the specific condition and other factors [20,21]. In this study, patients took over 120 min to reach the study site. Delays in referral and transfer lead to delays in diagnosis and management. The acute management of severe hypertension involves administering fast-acting antihypertensive agents, such as calcium channel blockers or alpha-beta blockers, which should be repeated after 30 min if the blood pressure remains above 160/110 [6]. Notably, only 5 out of the 12 patients with acute severe blood pressure readings at the referral centres received a fast-acting antihypertensive before transfer. Similarly, not all patients who required magnesium sulphate prophylaxis and long-acting antihypertensive medication received the necessary treatment. In one study, the mortality rate from eclampsia decreased by 46% and the risk of eclampsia was reduced by half in patients who received MgSO_4_ [22].

Emergency caesarean sections were performed in 80% of patients, and 20% had a normal vaginal delivery. The most common complications of HDPs in this study were renal dysfunction, HELLP syndrome, eclampsia, intracranial haemorrhage, and pulmonary oedema. A retrospective cohort study in Canada suggested that renal dysfunction in pre-eclamptic patients could be due to fluid-restrictive protocols and antihypertensive treatment [23]. In a study from Ethiopia looking at predictors of maternal mortality, elevated creatinine levels were a strong and independent predictor of maternal death [24]. A systematic review, also from Ethiopia, reported that complications mostly associated with HDPs include renal damage, pulmonary oedema, HELLP syndrome, and placental abruption [25]. In this study, 14 (63.6%) patients had HELLP syndrome, 10 (45.5%) had eclampsia, and 4 (18.2%) had intracranial haemorrhage.

The majority of the women died during the postpartum period. This finding is in keeping with other South African studies [5,12]. The final causes of death in our study population included eclampsia, renal failure, HELLP syndrome with disseminated intravascular coagulation (DIC), intracranial bleeding, pulmonary oedema, and pulmonary embolism. Schutte et al. identified cerebral haemorrhage as the leading cause of death among patients with HDPs, and most of the maternal deaths were associated with elevated systolic blood pressure and low platelet counts [26]. This finding is in contrast to a study conducted in Norway, which indicated that pulmonary oedema and intracranial haemorrhage were the most prevalent causes [15]. The variations in causes of death may reflect differences in disease severity, clinical resources, and local clinical practices. The most common patient-, administrative, and healthcare-related avoidable factors identified in this study were a lack of antenatal care, a lack of/or delay in emergency transport and, inappropriate management or response to abnormal clinical finding or results including a lack of resuscitation. Other studies have identified similar issues [5,12].

## 5. Conclusions

The majority of women who succumbed to hypertensive disorder of pregnancy-related complications were young Black Africans, and most of the maternal deaths occurred during the postpartum period. The most common avoidable factors included delays in ambulance response and a lack of adherence to protocols. Urgent attention must be directed towards addressing these factors to reduce HDP-associated maternal deaths at the study sites.

### 5.1. Strength and Limitations

Maternal deaths at the study site are reviewed within 24 h of their occurrence, ensuring that none of the maternal deaths were missed. The cause of death was supported by postmortem findings in the majority of cases. However, similarly to other retrospective studies, our research was limited by incomplete clinical records, primarily affecting referred patients.

### 5.2. Recommendations

We recommend implementing targeted interventions to improve early antenatal clinic booking and adherence to established clinical guidelines and protocols. Additionally, we advocate for the restoration of dedicated maternity ambulance services to mitigate the delays in ambulance response identified in this study. Furthermore, future research should focus on examining the factors that contribute to non-adherence to clinical guidelines and protocols across all levels of care.

## Figures and Tables

**Table 1:** Maternal characteristics of patients with HDPs.

Description (*n* = 23)	*n* (%) or Median (IQR; Range)
Age in years	27 (23–31; 18–39)
Body mass index (BMI)	33.5 (26.5–36.5; 19.5–49.9)
Previous mode of delivery	
Normal vaginal delivery (NVD)	9 (50.0%)
Caesarean section	3 (16.7%)
Median parity	1 (0–2; 0–2)
Booking status	
Booked	18 (78.2%)
Unbooked	5 (21.7%)
EGA at booking	15 (11.75–22.25; 6.0–30)
Co-morbidities (*n* = 8)	
Chronic hypertension (CHT)	3(37.5%)
Diabetes Mellitus (DM)	1(12.5%)
Previous gestational hypertensin (GHT)	4 (50.0%)
Prophylaxis	
Low-dose aspirin (LDA) initiated in women with risk factors at first booking presentation (*n* = 8)	1 (12.5%)

**Table 2:** Patient management at referring healthcare centres and study site.

Description	Referring Healthcare Centres (*n* = 18)	Study Site (*n* = 23)
	*n* (%) or Median (IQR; Range)	*n* (%) or Median (IQR; Range)
Blood pressure		
SBP (mmHg)	170(151–196; 130–241)	149.5 (137–183.0;101–238)
DBP	110(96–122; 80–163)	103.5 (89–123; 65–165)
Proteinuria		
Proteinuria on urine dipstick	18 (100)	19 (10.5%)
Spot urine protein-to-creatinine ratio (UCPCR) (mg/mmol)	0 (0.0%)	19 (90.5%)
Signs and symptoms of severity		
BP 160/110 or more	12 (66.7%)	16 (69.6%)
Eclamptic seizures	3 (16.7%)	6 (26.1%)
Frontal headache	5 (27.8%)	9 (39.1%)
Epigastric pain	3 (16.7%)	4(17.4%)
Visual disturbances	1 (5.6%)	2 (8.7%)
GCS less than 15	3 (16.7%)	7 (30.4%)
Management of acute elevated blood pressure (*n* = 12)		(*n* = 16)
Short-acting calcium channel blocker (Nifedipine^®^)	5 (41.7%)	7 (43.8%)
Alpha-beta blocker (Labetalol^®^) ^†^	0 (0.0%)	5 (31.3%)
Magnesium sulphate		
Loading dose	10 (83.3%)	13 (81.2%)
Maintenance dose	7 (58.3%)	9 (56.3%)
Long-acting/maintenance antihypertensive treatment		(*n* = 23)
First line	9 (75.0%)	15 (65.2%)
Second line	0 (0.0%)	4(17.4%)
Third line	0 (0.0%)	2 (8.7%)
Admission to obstetric high care (OHCU) and intensive care (ICU) units		
OHCU	0 (0.0%)	16 (73.9%)
ICU	0 (0.0%)	7 (30.4%)

^†^ No Labetalol or Hydralazine at the referring centres.

**Table 3:** Pregnancy outcomes and maternal complications.

Description (*n* = 23)	*n* (%) or Median (IQR; Range)
Delivery	
Estimated gestational age at delivery (*n* = 20)	32 (29–36, 20–39) weeks
NVD	4 (20.0%)
Caesarean section	16 (80.0%)
Maternal Complications of HDP (*n* = 23)	
Renal dysfunction	17 (73.9%)
HELLP syndrome	15 (65.2%)
Eclampsia	9 (39.1%)
Intracranial haemorrhage	4 (17.4%)
Pulmonary oedema	3 (13.0%)
Subcapsular liver haematoma	2 (8.7%)
Resuscitation	
Performed	16 (69.6%)
Not performed	7 (30.4%)
Brain dead	1 (14.3%)
Poor prognosis	1 (14.3%)
Reason(s) not stated	5 (71.4%)
Timing of deaths	
Postpartum	20 (87.0%)
Antepartum	3 (13.0%)
Place of Death	
General ward/obstetric admission area	4 (17.4%)
Obstetrics high care unit (OHCU)	7 (30.4%)
Intensive care unit (ICU)	12 (52.2%)
Day of death post-delivery (*n* = 20)	4 (1–8; 0–16)
Postmortem	
Performed	21 (91.3%)
Not performed	2 (8.7%)
Final cause of death	
Eclampsia	6 (26.1%)
Renal failure	6 (26.1%)
HELLP syndrome and DIC	5 (21.7%)
Intracranial bleed	4 (17.4%)
Pulmonary embolism	2 (8.7%)

**Table 4:** Avoidable factors.

Avoidable Factor (23)	*N* (%)
Patient-related	11 (47.5%)
Lack of antenatal care	5 (45.5%)
Late booking/inadequate	6 (54.5%)
Administrative	
Lack/delay in emergency transport	17 (73.9%)
Delay in finding an ICU bed/lack of ICU bed	11 (21.7%)
Healthcare-related	
Inappropriate management or response to abnormal clinical findings or results	12 (52.2%)
Lack of resuscitation	5 (21.7%)

## Data Availability

Anonymised data is available from the authors on request.
